# The *hokW-sokW* Locus Encodes a Type I Toxin–Antitoxin System That Facilitates the Release of Lysogenic Sp5 Phage in Enterohemorrhagic *Escherichia coli* O157

**DOI:** 10.3390/toxins13110796

**Published:** 2021-11-11

**Authors:** Kosuke Takada, Kotone Hama, Takaomi Sasaki, Yuichi Otsuka

**Affiliations:** Department of Biochemistry and Molecular Biology, Graduate School of Science and Engineering, Saitama University, 255 Shimo-Okubo, Sakura-Ku, Saitama 338-8570, Japan; t.kousuke080408@gmail.com (K.T.); k.hama.443@ms.saitama-u.ac.jp (K.H.); t.sasaki.675@ms.saitama-u.ac.jp (T.S.)

**Keywords:** bacteriophage, *Escherichia coli*, toxin–antitoxin system, prophage induction

## Abstract

The toxin-antitoxin (TA) genetic modules control various bacterial events, such as plasmid maintenance, persister cell formation, and phage defense. They also exist in mobile genetic elements, including prophages; however, their physiological roles remain poorly understood. Here, we demonstrate that *hokW-sokW*, a putative TA locus encoded in Sakai prophage 5 (Sp5) in enterohemorrhagic *Escherichia coli* O157: H7 Sakai strain, functions as a type I TA system. Bacterial growth assays showed that the antitoxic activity of *sokW* RNA against HokW toxin partially requires an endoribonuclease, RNase III, and an RNA chaperone, Hfq. We also demonstrated that *hokW-sokW* assists Sp5-mediated lysis of *E. coli* cells when prophage induction is promoted by the DNA-damaging agent mitomycin C (MMC). We found that MMC treatment diminished *sokW* RNA and increased both the expression level and inner membrane localization of HokW in a RecA-dependent manner. Remarkably, the number of released Sp5 phages decreased by half in the absence of *hokW-sokW*. These results suggest that *hokW-sokW* plays a novel role as a TA system that facilitates the release of Sp5 phage progeny through *E. coli* lysis.

## 1. Introduction

The toxin-antitoxin (TA) system is a genetic module composed of a toxin and an antitoxin. Genes encoding these elements are generally contiguous and have been discovered in almost all sequenced bacterial genomes [1]. While a toxin arrests cell growth, the antitoxin neutralizes its toxicity [2]. TA systems have been implicated in a wide range of bacterial events, such as plasmid maintenance [3], persister cell formation [4,5,6], and phage defense [7,8,9,10].

The first TA locus was discovered in the *E. coli* plasmid in 1983 [11,12]. Since then, TA loci have been found not only in plasmids but also on bacterial chromosomes [13,14,15]. TA loci are also closely linked to prophage genomes. For example, ~8% of the total TA loci have been overrepresented in the nine cryptic prophages of *E. coli* K-12 [16]. Cryptic prophages harbor mutations in genes required for virulence and phage formation, which render them trapped in the host genome. Although TA loci are also found in the genomes of functional prophages [17,18,19], the impact of their TA systems on the propagation of lysogenic phages remains poorly understood.

TA systems are currently classified into seven types according to the nature and function of antitoxins [20]. Type I antitoxins are small non-coding RNAs that bind to the cognate toxin mRNAs to inhibit translation or promote degradation [21,22]. The *hok-sok* locus encodes a type I TA system found in various plasmids and bacterial genomes. Hok (host killing) is a hydrophobic peptide toxin that depolarizes the plasma membrane and causes cell growth retardation and lysis [23,24]. *sok* (suppression of killing) is a small non-coding RNA that inhibits translation of *mok* (modulation of killing) mRNA that overlaps with *hok* mRNA. The base-pair RNAs formed between *sok* RNA and *mok* mRNA are degraded by RNase III that specifically cuts double-stranded RNA [25]. Since translation of *hok* and *mok* mRNAs is coupled, *sok* RNA indirectly inhibits the translation of *hok* mRNA [3]. While this TA system present in the plasmid has been implicated in plasmid maintenance [26] and phage defense [27], the roles of *hok-sok* loci encoded in prophage genomes remain unclear.

In this study, we aimed to clarify the role of a *hok-sok* locus encoded in a prophage of the enterohemorrhagic *E. coli* O157: H7 Sakai strain. Bacterial growth and survival assays and phage plaque formation experiments have provided evidence that supports a new role of the *hok-sok* TA system which facilitates the release of Sp5 phage progeny through *E. coli* lysis.

## 2. Results

### 2.1. hokW-sokW Functions as a TA System

**Figure 1:**
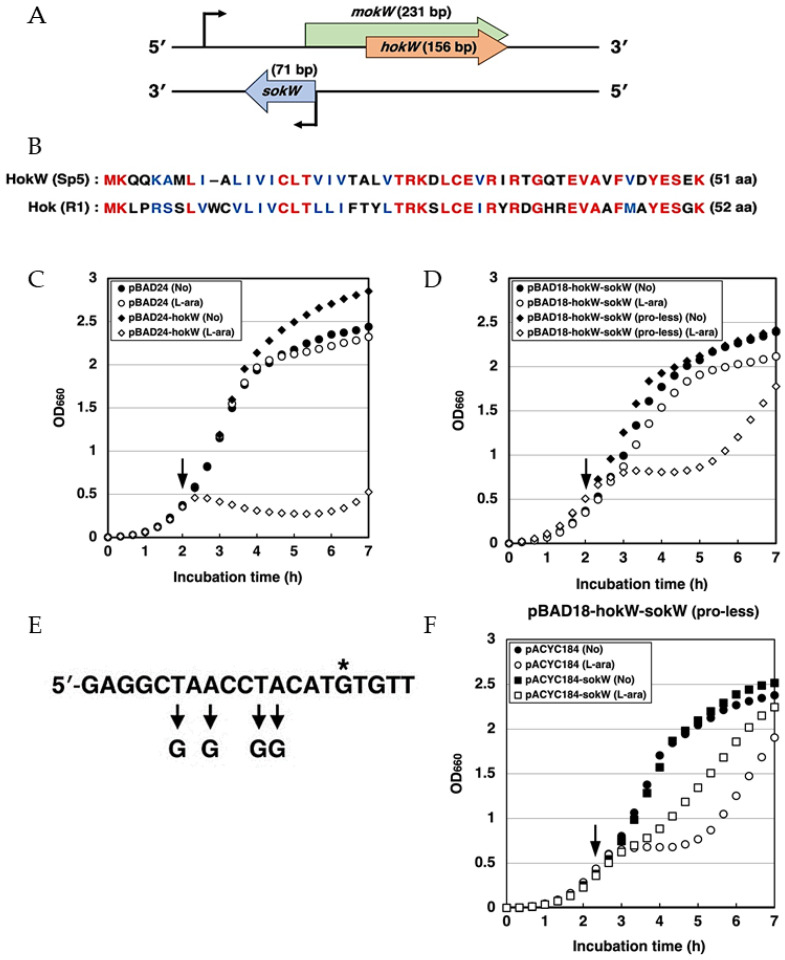
*hokW-sokW* functions as a TA system. (**A**) Genetic organization of *hokW-sokW* locus is shown. (**B**) Amino acid alignments of HokW from Sakai prophage 5 (Sp5) and Hok from *E. coli* R1 plasmid are shown. Red and blue letters indicate identical and similar residues, respectively. (**C**) TY0807 cells harboring pBAD24 or pBAD24-hokW were treated with or without L-arabinose (L-ara) when the OD_660_ reached 0.4 (indicated by the arrow) (**D**) TY0807 cells harboring pBAD18-hokW-sokW or pBAD18-hokW-sokW(pro-less) were treated with or without L-ara when the OD_660_ reached 0.4–0.5. (**E**) Nucleotide sequence around the –10 promoter element of *sokW* is shown. An asterisk is the transcription start site and arrows show nucleotides substituted in pBAD18-hokW-sokW(pro-less). (**F**) TY0807 cells harboring pBAD18-hokW-sokW(pro-less) plus either pACYC184-sokW or its vector pACYC184 were treated with or without L-ara when the OD_660_ reached 0.4.

*E. coli* O157: H7 Sakai strain has 18 prophages, three of which can infect non-pathogenic *E. coli* K-12 strains [28]. Among them, the lambdoid Sp5 (Sakai prophage 5) phage produces a major virulence factor called Shiga toxin 2. The genome of Sp5 phage encodes a gene pair named “*hokW-sokW*” that is predicted to be a member of the *hok*-*sok* family of type I TA system (Figure 1A) [29]. HokW (51 amino acids) shares 44% identity and 69% similarity with Hok (52 amino acids) in *E. coli* R1 plasmid (Figure 1B). We first examined whether *hokW-sokW* encoded in the Sp5 phage functions as a TA system. We constructed a plasmid pBAD24-hokW, in which a *hokW* is cloned under the control of an arabinose-inducible promoter. When HokW was expressed by arabinose in *E. coli* K-12 TY0807 cells, cell growth was immediately retarded (Figure 1C). Resumption of cell growth around 4 h after arabinose addition is probably due to the depletion of arabinose. Next, we tested whether *sokW* RNA suppressed the toxic activity of HokW. We cloned *hokW*-*sokW* in an arabinose-inducible plasmid, pBAD18-hokW-sokW, in which *hokW* mRNA is expressed from an arabinose-inducible promoter and *sokW* RNA from its own promoter. When TY0807 cells harboring this plasmid were treated with arabinose, no obvious growth retardation was observed (Figure 1D). To confirm the inhibitory effect of *sokW* RNA against HokW-mediated toxicity, we introduced mutations in the promoter sequence of *sokW* to repress its transcription (Figure 1E; pBAD18-hokW-sokW(pro-less)). TY0807 cells harboring pBAD18-hokW-sokW(pro-less) exhibited growth retardation for approximately 3 h after arabinose addition (Figure 1D). Importantly, the growth retardation observed in cells harboring pBAD18-hokW-sokW(pro-less) was partially restored by expressing *sokW* RNA using another plasmid, pACYC184-sokW (Figure 1F). These results strongly suggest that *sokW* RNA counteracts the toxic activity of HokW. Taken together, we concluded that HokW is a toxin and that *sokW* RNA functions as an antitoxin against HokW.

### 2.2. hokW-sokW Is a RNase III and Hfq Dependent Type I TA System

**Figure 2:**
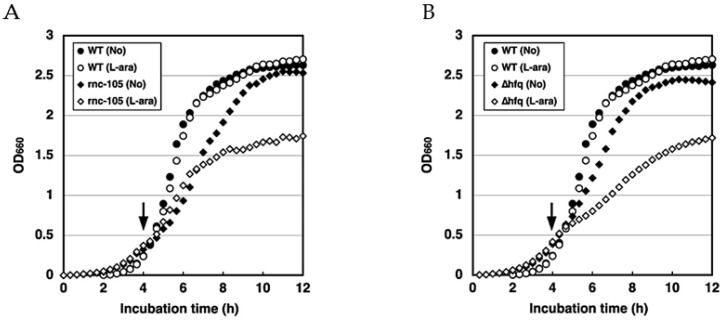
*hokW-sokW* is a RNase III and Hfq dependent type I TA system. (**A**) TY0807 (WT) or ME5413 (rnc-105) cells harboring pBAD18-hokW-sokW or (**B**) TY0807 (WT) or TY0807∆*hfq* (∆hfq) cells harboring pBAD18-hokW-sokW were treated with or without L-ara when the OD_660_ reached 0.4 (indicated by the arrow).

*sokW* RNA has a 71-nt complementary sequence to the 5′-UTR of *hokW* mRNA (Figure 1A). If *sokW* RNA forms a base pair in this region to repress *hokW* translation, it is likely that this process is RNase III-dependent [25]. To test this hypothesis, we performed cell growth assays with and without RNase III. While wild-type cells harboring pBAD18-hokW-sokW grew normally after arabinose addition, RNase III-deficient cells, ME5413, harboring the same plasmid exhibited growth retardation around 2 h after arabinose addition (Figure 2A). This result suggests the involvement of RNase III. RNase III-mediated double-strand RNA degradation is likely to play a crucial role in the antitoxic activity of *sokW* RNA. We next examined whether the antitoxic activity of *sokW* RNA requires Hfq, an RNA chaperone that facilitates base-pairing between the two RNAs [30]. When HokW expression was induced in *E. coli* cells lacking Hfq, cell growth was partially retarded despite the presence of *sokW* (Figure 2B). This result suggests that Hfq is involved in the antitoxic activity of *sokW* RNA and probably help base-pairing between *sokW* RNA and *hokW* mRNA. Together, these results suggest that *hokW-sokW* is a type I TA system whose antitoxin activity requires both RNase III and the Hfq chaperone.

### 2.3. hokW-sokW Assists Growth Retardation and Reduces Cell Viability after Mitomycin C Treatment

**Figure 3:**
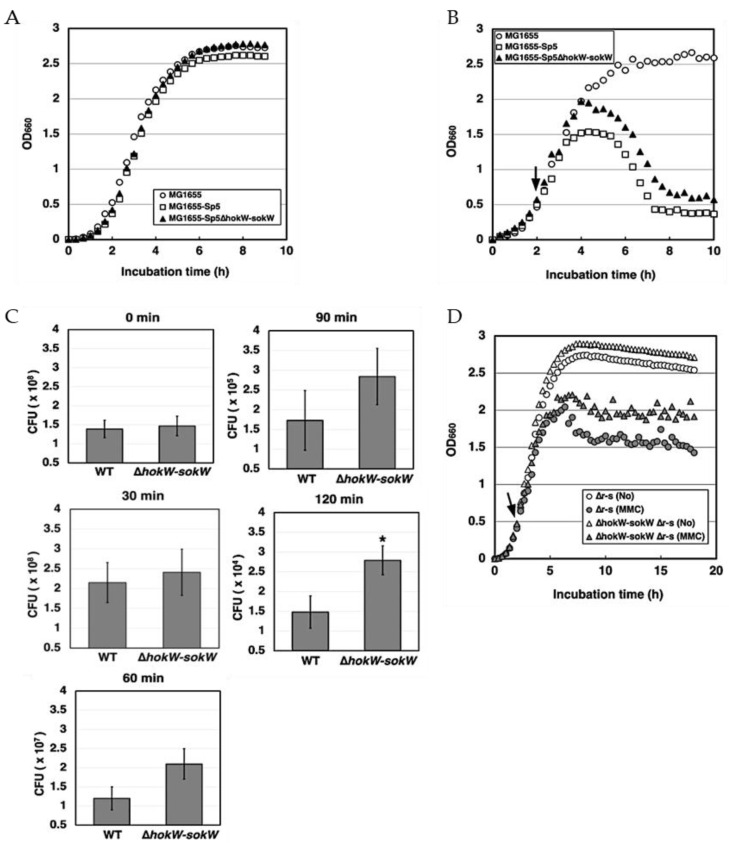
*hokW-sokW* assists the growth retardation and reduces cell viability after MMC treatment. (**A**) MG1655, MG1655-Sp5 or MG1655-Sp5(∆*hokW-sokW*) cells were grown in LB broth at 37 °C. (**B**) MG1655, MG1655-Sp5 or MG1655-Sp5(∆*hokW-sokW*) cells were treated with mitomycin C (MMC) when the OD_660_ reached to 0.5 (indicated by the arrow). (**C**) MG1655-Sp5 (WT) or MG1655-Sp5(∆*hokW-sokW*) (∆*hokW-sokW*) cells were grown until the OD_660_ reached to 0.4 and then treated with MMC for 0, 30, 60, 90, and 120 min. The colony-forming unit (CFU) data represents the mean ± standard deviation (SD) of at least triplicate measurements. * *p* < 0.05 versus WT cells. (**D**) MG1655-Sp5(∆*r-s*) (∆r-s) or MG1655-Sp5(∆*hokW-sokW* ∆*r-s*) (∆hokW-sokW ∆r-s) cells were treated with or without MMC when the OD_660_ reached to 0.4–0.5.

*hokW-sokW* is encoded in the lysogenic Sp5 prophage of the *E. coli* O157: H7 Sakai strain. To explore the potential role of *hokW-sokW* in Sp5-mediated cell growth retardation, we compared cell growth and viability after Sp5 prophage induction with or without this type I TA system. For the experiment, *E. coli* K-12 MG1655 cells with Sp5 prophage were used. We first confirmed that deletion of *hokW-sokW* did not affect cell growth under normal conditions (Figure 3A). The Sp5 prophage was induced in MG1655 cells using mitomycin C (MMC), a DNA-damaging agent that crosslinks two DNA bases and triggers prophage induction [31]. MMC is one of the most commonly used antibiotics for prophage induction. While MMC had no obvious effect on MG1655 cell growth, Sp5-containing cells (MG1655-Sp5) stopped growing around 2 h after MMC treatment and started to lyse around 3.5 h (Figure 3B). Remarkably, growth retardation started approximately 40 min later in the absence of *hokW-sokW* (MG1655-Sp5(∆*hokW*-*sokW*)), indicating that this TA system facilitates Sp5-mediated growth retardation. To assess whether *hokW-sokW* also affects cell survival, we measured the number of viable cells at different time points after MMC treatment. At 30 min after the treatment, the cell viability of MG1655-Sp5 was comparable to that of MG1655-Sp5(∆*hokW*-*sokW*). However, the viability of MG1655-Sp5 dropped to about 55% of that of MG1655-Sp5(∆*hokW*-*sokW*) for the cells harvested 120 min (Figure 3C). These results indicate that *hokW-sokW* not only facilitates growth retardation but also reduces cell survival.

HokW toxin is predicted to be a short peptide that destabilizes the plasma membrane. To examine whether *hokW-sokW*-mediated growth retardation is dependent on the lytic enzymes encoded in the Sp5 phage, we created a prophage that lacks both the R endolysin and the S holin (MG1655-Sp5(∆*r-s*)). The growth retardation of MG1655-Sp5(∆*r-s*) started approximately 3 h after MMC treatment, likely due to the production of Sp5 phages in the cells (Figure 3D). Notably, the retardation of cell growth was less severe in the absence of *hokW-sokW* (MG1655-Sp5(∆*hokW-sokW* ∆*r-s*)). These results strongly suggest that *hokW-sokW* promotes growth retardation and possibly cell lysis independent of the lytic enzymes encoded in the Sp5 phage.

### 2.4. MMC Triggers HokW Toxin Production in a RecA Dependent Manner

**Figure 4:**
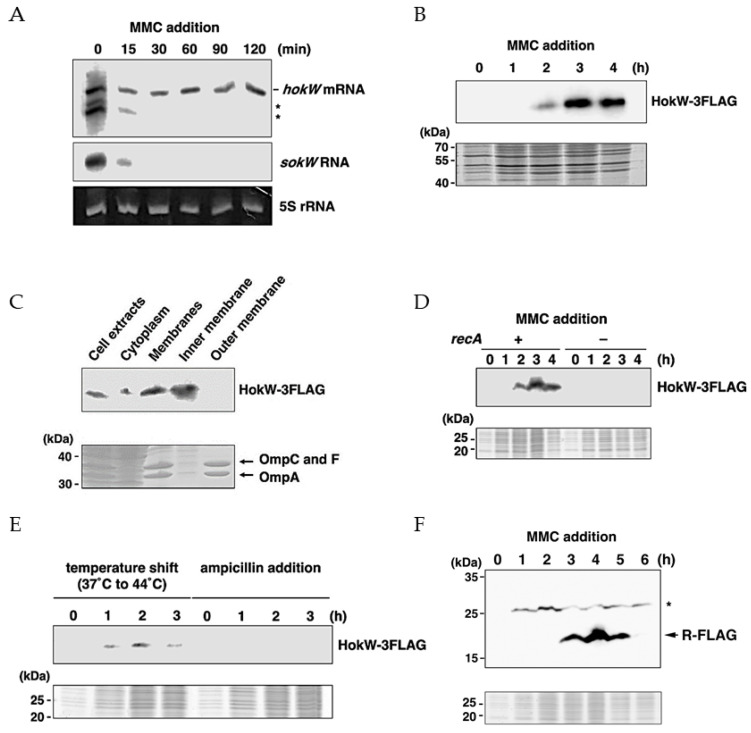
MMC triggers HokW toxin production in an RecA-dependent manner. (**A**) Total RNA was extracted from MG1655-Sp5 cells at the indicated times after MMC addition and subjected to northern blotting with oligo-probes for *hokW* mRNA and *sokW* RNA. Ethidium bromide-stained 5S rRNA was used as a loading control. Asterisks indicate bands corresponding to truncated *hokW* RNAs or non-specific bands. (**B**) Total proteins extracted from MG1655-Sp5(*hokW*-*3FLAG*) cells at the indicated times after MMC addition were subjected to western blotting with an anti-FLAG antibody (upper panel). Coomassie Brilliant Blue (CBB)-stained proteins were used as loading controls (bottom panel). (**C**) MG1655-Sp5(*hokW*-*3FLAG*) cells were treated with MMC for 2 h and cell lysates were fractionated as described in Materials and Methods. The upper and lower panels show the western blot or the CBB staining. OmpA, C, and F are the outer membrane proteins. Many proteins were detected in the CBB staining of cell extracts and cytoplasm fraction because of concentration to get a signal in the western blotting. (**D**) Total proteins extracted from MG1655-Sp5(*hokW*-*3FLAG*) (+) or MG1655 ∆*recA*-Sp5(*hokW*-*3FLAG*) (–) cells were subjected to western blotting and CBB staining. (**E**) MG1655-Sp5(*hokW*-*3FLAG*) cells were shifted from 37 °C to 44 °C or treated with 1 µg/mL ampicillin, and total proteins were subjected to western blotting and CBB staining. (**F**) Total proteins extracted from MG1655-Sp5(*r-FLAG*) cells were subjected to western blotting and CBB staining. The asterisk indicates a non-specific band.

These results suggest that MMC treatment induces HokW toxin expression. To examine this possibility, we first compared the amounts of *hokW* mRNA and *sokW* RNA before and after MMC treatment. As shown in Figure 4A, both *hokW* mRNA and *sokW* RNA were detected before MMC treatment, indicating that MMC does not promote *hokW* transcription. While *hokW* mRNA remained stable after MMC treatment, *sokW* RNA rapidly disappeared. These results suggest that HokW is produced after MMC treatment. Next, we measured the amount of HokW toxin using western blotting. Production of a FLAG-tagged HokW (HokW-3FLAG) started 2 h after MMC treatment and plateaued around 3 h (Figure 4B). Fractionated bacterial samples showed that HokW-3FLAG was enriched in the inner membrane, as seen with other members of the Hok family toxins (Figure 4C) [23]. These results suggest that MMC treatment promotes HokW production and localization to the inner membrane through the disappearance of *sokW* RNA.

It is well known that treating lysogenic *E. coli* with a DNA-damaging agent such as MMC activates RecA, which stimulates the self-cleavage of the CI repressor to initiate prophage induction [32]. RecA is a single-stranded DNA-binding protein that is involved in DNA recombination and repair [33]. To examine whether HokW production triggered by MMC requires RecA, we constructed a deletion construct of *recA* in MG1655-Sp5(*hokW-3FLAG*) and measured the level of HokW (Figure 4D). Western blotting revealed that HokW-3FLAG was not produced in ∆*recA* cells even 4 h after MMC treatment. This indicates that HokW production is RecA-dependent. HokW-3FLAG production was also observed when cells were shifted from 37 °C to 44 °C to trigger prophage induction (Figure 4E). Notably, HokW-3FLAG was not produced when cells were treated with ampicillin that does not trigger prophage induction. These results strongly suggest that RecA-mediated prophage induction causes HokW production.

Finally, we examined the expression timing of HokW and the R endolysin after MMC treatment. Western blotting showed that HokW production started approximately 1 h earlier than the R (Figure 4B,F). This result suggests that HokW-mediated growth retardation and possibly cell lysis precedes cell lysis mediated by lytic enzymes, the R endolysin and the S holin, of Sp5 phage.

### 2.5. hokW-sokW Facilitates the Release of Sp5 Phage Progeny

**Figure 5:**
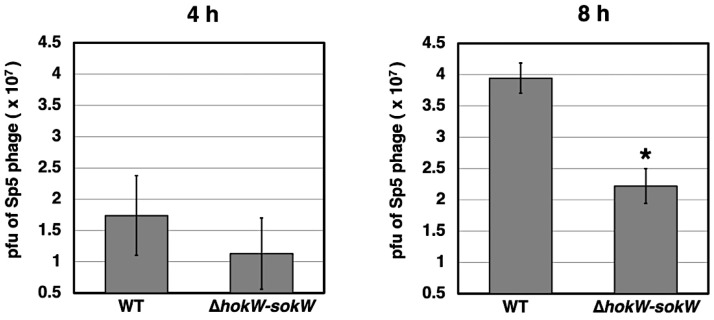
*hokW-sokW* facilitates the release of Sp5 phage progeny. MG1655-Sp5(km^r^) (WT) or MG1655-Sp5(∆*hokW-sokW*) (∆*hokW-sokW*) cells were grown and then treated with MMC for 4 h or 8 h. Sp5 phages released were plated with MG1655 cells to measure plaque-forming units (pfu). The pfu data represent the mean ± SD of at least triplicate measurements. * *p* < 0.05, versus WT cells.

Finally, we assessed whether the *hokW-sokW* TA system plays a role in phage propagation. We measured the number of Sp5 phage progeny released from MG1655 cells. Compared to wild-type, Sp5 phage progenies released from ∆*hokW-sokW* cells at 8 h after MMC treatment were reduced by around half (Figure 5). This suggests that *hokW-sokW* facilitates the release of Sp5 phage progeny.

## 3. Discussion

TA loci are abundant in bacterial genomes and mobile genetic elements and are involved in various bacterial events, such as plasmid maintenance, persister cell formation, and phage defense. Here, we addressed the physiological role of a putative TA module encoded in the genome of a functional prophage in the *E. coli* O157: H7 Sakai strain.

We first demonstrated that *hokW-sokW*, encoded in the lysogenic Sp5 phage, functions as a type I TA system (Figure 1 and Figure 2). When cells are exposed to extracellular stresses such as phage infection, type II TA genes are transcriptionally repressed, and consequently, labile antitoxins disappear and the toxins are activated [9,10,34]. In the *hokW-sokW* TA system, *sokW* RNA disappeared immediately after MMC treatment, and the remaining *hokW* mRNA was translated (Figure 4A,B). Considering that MMC damages DNA, the disappearance of *sokW* RNA would be caused by blocking *sokW* transcription rather than augmenting mRNA degradation activity. MMC-mediated DNA damage results in the activation of RecA and initiation of prophage induction [31,32]. Intriguingly, HokW expression requires RecA (Figure 4D). Thus, prophage induction rather than DNA damage itself is likely to trigger the production of HokW toxin. This idea is also supported by the result of Figure 4E. Prophage induction involves many biological processes, such as excision, cyclization, replication of phage DNA, expression of phage proteins, and assembly of phage particles. Further studies are required to clarify which of these processes lead to the loss of *sokW* RNA and HokW production.

Previous reports demonstrated that *hok* mRNA from *E. coli* R1 plasmid is slowly processed at its 3′-end after the transcription is stopped by rifampicin [3]. This truncated *hok* RNA is translated more actively than full-length *hok* mRNA [35]. In Figure 4A, two smaller RNAs than full-length *hokW* mRNA were detected and rapidly disappeared after MMC addition. Whether these RNAs correspond to the truncated *hok* RNA from R1 plasmid needs to be investigated. Although RNase III and Hfq were suggested to be required for *sokW* RNA-mediated translational repression of *hokW* mRNA, this antitoxic activity was partially effective in the RNase III and Hfq defective constructs (Figure 2). Therefore, RNase III and Hfq are unlikely to play a major role in *the sokW* RNA-mediated translational repression. In line with this idea, neither RNase III nor Hfq mutants are lethal. Presumably, there is another mechanism for repressing HokW expression besides the cleavage of *hokW* mRNA by RNase III. And the base-pairing between *hokW* mRNA and *sokW* RNA would naturally occur in the absence of Hfq, albeit inefficiently.

**Figure 6:**
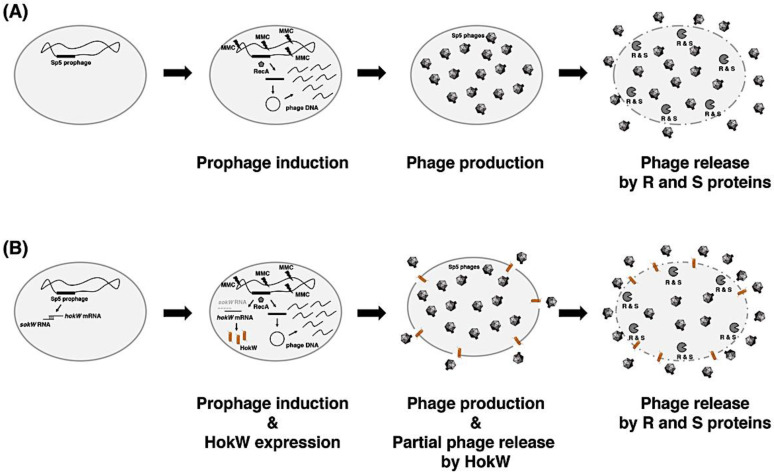
Working hypothesis for the function of *hokW-sokW* in Sp5 phage propagation. (**A**) When Sp5 prophage does not encode the *hokW-sokW* TA locus, MMC treatment triggers the initiation of Sp5 prophage induction through RecA activation. After Sp5 phage DNA is excised from the host genome, cyclized, and amplified, Sp5 phage particles are produced. Lytic enzymes (Holin; S and Endolysin; R) are expressed at the late stage after induction, which results in cell lysis and the release of phage progeny. (**B**) When Sp5 prophage encodes *hokW-sokW*, MMC treatment triggers not only Sp5 prophage induction but also HokW production. HokW localizes to the inner membrane and partially causes cell lysis before lytic enzymes are fully expressed. Consequently, Sp5 phage progenies are released earlier than usual.

The type I TA toxins are classified into two types: membrane-associated toxins and cytosolic toxins [36]. The membrane-associated toxins are small hydrophobic peptides that localize in the inner membrane, which affects the plasma membrane integrity and potential, and consequently leads to growth retardation and cell lysis [37,38]. Similarly, HokW are hydrophobic peptides (Figure 1B) and are localized in the inner membrane (Figure 4C). As expected, endogenous HokW production triggered by MMC facilitated growth retardation (Figure 3B,D) and decreased cell viability (Figure 3C). These results suggested that cell lysis would begin before the production of phage progeny is completed and that the number of phage progenies released would be decreased. However, contrary to this prediction, cells harboring *hokW-sokW* released phage progenies approximately 2-fold compared to the ∆*hokW-sokW* cells (Figure 5). Based on these results, we propose a working hypothesis in which *hokW-sokW* plays a positive role in Sp5 phage propagation. If the Sp5 prophage does not encode the *hokW-sokW* TA locus, MMC treatment triggers the initiation of Sp5 prophage induction through RecA activation. Lysis enzymes are expressed at the late stage after induction, which results in cell lysis and the release of phage progeny (Figure 6A). If the Sp5 prophage encodes *hokW-sokW*, MMC treatment triggers not only Sp5 prophage induction but also HokW production. HokW localizes to the inner membrane and partially causes cell lysis before lysis enzymes are fully expressed. Consequently, the release of Sp5 phage progeny starts earlier than usual (Figure 6B).

The RnlA-RnlB TA system present in the cryptic CP4-57 prophage of the *E. coli* K-12 strain inhibits the propagation of lytic bacteriophage T4 [9]. Other TA systems (antiQ-AbiQ, ToxIN, MazE-MazF, etc.) have also been reported to function in defense against lytic phages [7,8,34,39]. Therefore, TA loci in the bacterial genome are believed to function as a mechanism that inhibits phage propagation. However, this study demonstrates that a TA system encoded in a lysogenic prophage has a positive effect on phage propagation, which is a new physiological role of TA systems. To date, the *hok-sok* TA system present in the R1 plasmid has been reported to inhibit T4 phage propagation [27]. In *E. coli* K-12 strain harboring a high-copy plasmid containing *hok-sok*, the efficiency of plating of T4 phage was reduced by 42% and plaque size was decreased by ~85%. The molecular mechanism of phage inhibition by this *hok-sok* TA system remains unclear. Interestingly, TA systems belonging to the same family play opposite physiological roles in phage propagation depending on the type of mobile genetic element where the TA loci are located.

## 4. Materials and Methods

### 4.1. E. coli Strains

The *E. coli* strains and the primers (eurofins, Tokyo, Japan) used in this study are listed in Table 1 and Table 2. All *E. coli* strains used in this study belong to non-pathogenic *E. coli* K-12 strain. MG1655, MG1655-Sp5, and MG1655-Sp5(Km^r^) were kindly gifted by Prof. Sekine at Rikkyo University [40]. MG1655-Sp5 and its derivatives do not produce Shiga toxin 2 because of the deletion of *stx2A-stx2B*. MG1655-Sp5(∆*hokW-sokW*::*km^r^*), MG1655-Sp5(∆*r-s*::*cat*), and MG1655-Sp5(∆*hokW-sokW*::*km^r^* ∆*r-s*::*cat*) were constructed as described previously [41]. Briefly, a fragment containing a kanamycin-resistant or a chloramphenicol-resistant cassette flanked with the sequences upstream and downstream of the deleted genes was amplified by polymerase chain reaction (PCR) with pKD4 or pKD3 as a template. Primers YO-351 and YO-352 for *hokW-sokW*::*km^r^*, or YO-635 and YO-636 for *r-s*::*cat* were used. The amplified fragment was inserted into MG1655-Sp5 or MG1655-Sp5(∆*hokW-sokW*::*km^r^*) harboring pKD46 encoding λ phage Red recombinase, and kanamycin-resistant or chloramphenicol-resistant colonies were screened by PCR with primers YO-349 and YO-350, or YO-637 and YO-638. MG1655-Sp5(*hokW-3FLAG-km^r^*) and MG1655-Sp5(*r-FLAG-cat*) were constructed as previously described [42]. Briefly, a fragment containing a kanamycin-resistant or a chloramphenicol-resistant cassette was amplified by PCR with the pSUP11 template and primers YO-560 and YO-561 for *hokW-3FLAG*-*km^r^* or the pSU313 template and primers YO-644 and YO-657 for *r*-*FLAG*-*cat*. The amplified fragments were inserted into MG1655-Sp5 harboring pKD46 and kanamycin-resistant or chloramphenicol-resistant colonies were screened by PCR with primers YO-349 and YO-350, or YO-637 and YO-638. To eliminate the kanamycin-resistant cassette, MG1655-Sp5(*hokW-3FLAG*-*km^r^*) was transformed with pCP20 that causes temperature-sensitive replication and thermal induction of FLP synthesis. MG1655-Sp5(*hokW-3FLAG*) was selected by PCR using primers YO-349 and YO-350. TY0807 ∆*hfq*::*km^r^* and MG1655 ∆*recA*::*km^r^*-Sp5(*hokW*-*3FLAG*) were constructed by GT7 phage-mediated transduction of a kanamycin-resistance cassette from JW4130 and JW2669, respectively [43].

### 4.2. Plasmid Construction

To generate pBAD24-hokW or pBAD18-hokW-sokW, a DNA fragment containing *hokW* or *hokW*-*sokW* was amplified by PCR using MG1655-Sp5 DNA as a template and primers YO-357 and YO-369, or YO-432 and YO-433, digested with *Eco*RI (NIPPON GENE, Tokyo, Japan) and *Pst*I (NIPPON GENE, Tokyo, Japan) or *Eco*RI and *Hin*dIII (NIPPON GENE, Tokyo, Japan), and ligated into the corresponding sites of pBAD24 or pBAD18 [44]. The promotor mutant construct, pBAD18-hokW-sokW(pro-less), was created using the KOD-Plus-Mutagenesis kit (TOYOBO, Osaka, Japan) with primers YO-421 and YO-422 and pBAD18-hokW-sokW as a template. To construct pACYC184-sokW, a DNA fragment containing *sokW* was amplified by PCR using MG1655-Sp5 DNA as a template and primers YO-406 and YO-541, digested with *Bam*HI (NIPPON GENE, Tokyo, Japan) and *Hin*dIII, and ligated into the corresponding sites of pACYC184. DNA sequences of the constructed plasmids were confirmed by sequencing.

### 4.3. E. coli Growth and CFU Assay

*E. coli* cells were grown in Luria–Bertani (LB) broth or LB broth supplemented with ampicillin (nacalai tesque, Kyoto, Japan) or chloramphenicol (nacalai tesque, Kyoto, Japan) at 37 °C. When the optical density at 660 nm (OD_660_) reached 0.3–0.5, 0.2% L-arabinose (L-ara) (FUJIFILM Wako Pure Chemical, Osaka, Japan) was added to express the protein from the pBAD plasmids or 2.0 µg/mL mitomycin C (MMC) (FUJIFILM Wako Pure Chemical, Osaka, Japan) was added to induce Sp5 prophage. Cell densities were monitored every 20 min using a biophotorecorder (ADVANTEC TVS062CA, Tokyo, Japan). At least triplicate measurements were performed, and similar results were obtained for each measurement. A representative result is shown in each figure. In colony-forming unit (CFU) assay, *E. coli* cells were grown in LB broth at 37 °C until the OD_660_ reached 0.4, and harvested at 0, 30, 60, 90, and 120 min after 2.0 µg/mL MMC addition. Cells were diluted with phosphate-buffered saline (PBS) (nacalai tesque, Kyoto, Japan), plated onto LB agar plates, and incubated at 37 °C overnight. The colonies that emerged were counted and the number of viable cells in 1 mL culture medium was calculated.

### 4.4. Northern Blotting Analysis

MG1655-Sp5 cells were grown in LB broth at 37 °C until the OD_660_ reached 0.4, and harvested at 0, 15, 30, 60, 90, and 120 min after 2.0 µg/mL MMC addition. Total RNA was isolated and purified as previously described [45]. Total RNA (2.0 µg) was electrophoresed on a 6% polyacrylamide gel (FUJIFILM Wako Pure Chemical, Osaka, Japan) containing 7 M urea (FUJIFILM Wako Pure Chemical, Osaka, Japan), followed by northern blotting. The digoxigenin (Dig)-labeled oligo-probe YO-597 or YO-592 (eurofins, Tokyo, Japan) was used for *hokW* mRNA or *sokW* RNA. After hybridization, the membranes were probed with anti-digoxigenin-AP Fab fragments (Roche, Basel, Switzerland), and RNAs were detected using CDP-Star chemiluminescent substrate (Roche, Basel, Switzerland) and the C-DiGit Blot Scanner (LI-COR, Lincoln, NE, USA).

### 4.5. Fractionation of Cell Extracts and Western Blotting Analysis

*E. coli* cells were grown in LB broth at 37 °C until the OD_660_ reached 0.4, harvested at appropriate times after 2.0 µg/mL MMC addition, and resuspended in PBS. For fractionation, MG1655-Sp5(*hokW-3FLAG-km^r^*) cells were grown in 20 mL LB broth supplemented with kanamycin (nacalai tesque, Kyoto, Japan) at 37 °C and harvested at 2 h after 2.0 µg/mL MMC addition. Cells were resuspended in 0.8 mL PBS and lysed by sonication. After cell debris was removed, the resulting supernatant (0.7 mL) was centrifuged at 20,000× *g* for 45 min at 4 °C to separate the cytoplasmic and membrane fractions. To separate the inner and outer membranes, the pellet containing membrane fraction was resuspended in 0.35 mL PBS containing 0.4% Sarcosyl (nacalai tesque, Kyoto, Japan) and incubated for 30 min at room temperature [46]. This mixture was centrifuged at 20,000× *g* for 30 min at 4 °C, and the supernatant was used as the inner membrane fraction. The pellet was resuspended in 0.35 mL PBS and used as the outer membrane fraction. Proteins were separated on 12.5% or 18% polyacrylamide gels (FUJIFILM Wako Pure Chemical, Osaka, Japan) containing sodium dodecyl sulfate (FUJIFILM Wako Pure Chemical, Osaka, Japan) for western blotting and Coomassie Brilliant Blue (CBB) (nacalai tesque, Kyoto, Japan) staining. For detection of FLAG-tagged proteins, proteins were electroblotted onto an Amersham Hybond P PVDF 0.2 membrane (Cytiva, Tokyo, Japan), probed with a mouse anti-FLAG M2 monoclonal antibody (Sigma-Aldrich, St. Louis, MO, USA) and with a horseradish peroxidase-conjugated sheep anti-mouse IgG (GE Healthcare, Chicago, IL, USA). Chemi-Lumi One Ultra (nacalai tesque, Kyoto, Japan) or Amersham ECL Prime (GE Healthcare, Chicago, IL, USA) were used as substrates for detection with the C-DiGit Blot Scanner (LI-COR).

### 4.6. Plaque Formation Assay of Sp5 Phages

MG1655-Sp5(km^r^) or MG1655-Sp5(∆*hokW*-*sokW*) cells were grown in LB broth supplemented with kanamycin at 37 °C until the OD_660_ reached 0.4 and treated with 2.0 µg/mL MMC for 4 or 8 h. Cell cultures were centrifuged at 8000× *g* for 3 min and the supernatant was used for plaque formation assay. The phage solution was diluted and mixed with MG1655 as an indicator cell in soft agar containing LB broth, 0.3% agar (FUJIFILM Wako Pure Chemical, Osaka, Japan), 1.5 µg/mL MMC and 10 mM CaCl_2_ (FUJIFILM Wako Pure Chemical, Osaka, Japan). The mixture was poured onto LB-agar plate and incubated at 37 °C overnight. The plaques that emerged on the plate were counted and the number of phages in 1 mL phage solution was calculated for the plaque-forming units (pfu).

## Figures and Tables

**Table 1:** *Escherichia coli* strains used in this study.

Strains	Genotype	Source/Reference
TY0807	*sup^0^ araD139 hsdR* ∆*lacX74 rpsL araD^+^*	[9]
TY0807 ∆*hfq*	TY0807 ∆*hfq*::*km^r^*	This study
BW25113	*rrnB3* ∆*lacZ4787 hsdR514* ∆*(araBAD)567* ∆*(rhaBAD)568 rph-1*	NBRP-*E. coli* at NIG
JW2669	BW25113 ∆*recA*::*km^r^*	NBRP-*E. coli* at NIG
JW4130	BW25113 ∆*hfq*::*km^r^*	NBRP-*E. coli* at NIG
ME5413	*metB1 his rnc-105 ranA2074*	NBRP-*E. coli* at NIG
MG1655	λ^-^ *ilvG^-^ rfb-50 rph-1*	[40]
MG1655-Sp5	MG1655-Sp5(∆*stx2AB*)	[40]
MG1655-Sp5(km^r^)	MG1655-Sp5(∆*stx2AB*::*km^r^*)	[40]
MG1655-Sp5(∆*hokW*-*sokW*)	MG1655-Sp5(∆*stx2AB* ∆*hokW*-*sokW*::*km^r^*)	This study
MG1655-Sp5(∆*r-s*)	MG1655-Sp5(∆*stx2AB* ∆*r-s*::*cat*)	This study
MG1655-Sp5(∆*hokW*-*sokW* ∆*r-s*)	MG1655-Sp5(∆*stx2AB* ∆*hokW*-*sokW*::*km^r^* ∆*r-s*::*cat*)	This study
MG1655-Sp5(*hokW*-*3FLAG-km^r^*)	MG1655-Sp5(∆*stx2AB hokW*-*3FLAG*-*km^r^*)	This study
MG1655-Sp5(*hokW*-*3FLAG*)	MG1655-Sp5(∆*stx2AB hokW*-*3FLAG*)	This study
MG1655 ∆*recA*-Sp5(*hokW*-*3FLAG*)	MG1655 ∆*recA*::*km^r^*-Sp5(∆*stx2AB hokW*-*3FLAG*)	This study
MG1655-Sp5(*r*-*FLAG*)	MG1655-Sp5(∆*stx2AB r*-*FLAG-cat*)	This study

**Table 2:** The oligonucleotides used in this study.

Primer Name	Sequence (5′–3′)
YO-349	CTTGAGGCTATCTGCCTCGGGCATG
YO-350	GCGTTGAGGATGCCTGACACATCAG
YO-351	GCGGGTGCTTGAGGCTATCTGCCTCGGGCATGAACACCAACGGCAGATAGCATATGAATATCCTCCTTAG
YO-352	CACATCAGAGGTGGCGGGAGATTACTCCCCCGCTTGGTCTCTTACTTCTCGTGTAGGCTGGAGCTGCTTC
YO-357	AGGAATTCACCATGAAGCAGCAAAAGGCGATG
YO-369	TCCTGCAGTTACTTCTCAGATTCGTAGTC
YO-406	TCAAGCTTTCATAGCCTGCTTCTCCTTGCC
YO-421	CCGGCTCGCCTCTTACGTGCCGAAAG
YO-422	CATGTGTTCAGCATGGATTGAGCCTC
YO-432	AGGAATTCGAATCAATGACCTGGCCTGAAGC
YO-433	AGAAGCTTGAAATAAGTGCTGCAATCAATAC
YO-541	AGGGATCCGCCTCGGGCATGAACACCAAC
YO-560	GAACCGGTCAGACGGAGGTCGCTGTCTTCGTAGACTACGAATCTGAGAAGGACTACAAAGACCATGACGG
YO-561	TCAGAGATGAACATTCAAACAGCATTTTCAGTATGGTAAAGCGCGGGTGCCATATGAATATCCTCCTTAG
YO-592	[Dig]-CATTAATCTGAGGCTCAATCCATGCTGAAC
YO-597	[Dig]-CCTTGCCTTTCGGCACGTAAGAGGCTAACC
YO-635	ACAGCTGCTGGCCTTTTTCATGTTGTGAGCTTCCGGATTGCGGGAGACGGGTGTAGGCTGGAGCTGCTTC
YO-636	TTCGTGTTATCCGTCCATGTAAGCAAACCTCATTTT TCAGCAAAATATTCCATATGAATATCCTCCTTAG
YO-637	CCCGAATCGGTCATGATGCTGTAAC
YO-638	AACTGTTTTGACTTTATTCACTTAC
YO-644	TTTTGACTTTATTCACTTACATTTTGCCAATTTGCAGGATTTCGTGTTATCATATGAATATCCTCCTTAG
YO-657	GTATCCCGTCGTGACCAGGAGAGCGCGCTGGCGTGCTGGGGAATCGACAGAGACTACAAAGATGACGACG

## Data Availability

All data for this study are available within the article.
